# A Trimodal Imaging Platform for Tracking Viable Transplanted Pancreatic Islets *In Vivo*: F-19 MR, Fluorescence, and Bioluminescence Imaging

**DOI:** 10.1007/s11307-018-1270-3

**Published:** 2018-08-30

**Authors:** A. Gálisová, V. Herynek, E. Swider, E. Sticová, A. Pátiková, L. Kosinová, J. Kříž, M. Hájek, M. Srinivas, D. Jirák

**Affiliations:** 1 MR Unit, Department of Radiodiagnostic and Interventional Radiology, Institute for Clinical and Experimental Medicine, Prague, Czech Republic; 2 Center for Advanced Preclinical Imaging, First Faculty of Medicine, Charles University, Prague, Czech Republic; 3 Department of Tumor Immunology, Radboud University Medical Center, Nijmegen, Netherlands; 4 Department of Clinical and Transplant Pathology, Institute for Clinical and Experimental Medicine, Prague, Czech Republic; 5 Department of Pathology, Third Faculty of Medicine, Charles University, Prague, Czech Republic; 6 Centre of Experimental Medicine, Institute for Clinical and Experimental Medicine, Prague, Czech Republic; 7 Diabetes Centre, Institute for Clinical and Experimental Medicine, Prague, Czech Republic; 8 Institute of Biophysics and Informatics, First Faculty of Medicine, Charles University in Prague, Prague, Czech Republic

**Keywords:** F-19 magnetic resonance imaging, Optical imaging, Pancreatic islets, Transplantation, Nanoparticles

## Abstract

**Purpose:**

Combining specific and quantitative F-19 magnetic resonance imaging (MRI) with sensitive and convenient optical imaging provides complementary information about the distribution and viability of transplanted pancreatic islet grafts. In this study, pancreatic islets (PIs) were labeled with positively charged multimodal nanoparticles based on poly(lactic-co-glycolic acid) (PLGA-NPs) with encapsulated perfluoro-15-crown-5-ether and the near-infrared fluorescent dye indocyanine green.

**Procedures:**

One thousand and three thousand bioluminescent PIs were transplanted into subcutaneous artificial scaffolds, which served as an alternative transplant site. The grafts were monitored using *in vivo* F-19 MR, fluorescence, and bioluminescence imaging in healthy rats for 2 weeks.

**Results:**

Transplanted PIs were unambiguously localized in the scaffolds by F-19 MRI throughout the whole experiment. Fluorescence was detected in the first 4 days after transplantation only. Importantly, *in vivo* bioluminescence correlated with the F-19 MRI signal.

**Conclusions:**

We developed a trimodal imaging platform for *in vivo* examination of transplanted PIs. Fluorescence imaging revealed instability of the fluorescent dye and its limited applicability for longitudinal *in vivo* studies. A correlation between the bioluminescence signal and the F-19 MRI signal indicated the fast clearance of PLGA-NPs from the transplantation site after cell death, which addresses a major issue with intracellular imaging labels. Therefore, the proposed PLGA-NP platform is reliable for reflecting the status of transplanted PIs *in vivo*.

**Electronic supplementary material:**

The online version of this article (10.1007/s11307-018-1270-3) contains supplementary material, which is available to authorized users.

## Introduction

Intrahepatic transplantation of pancreatic islets (PIs) represents an alternative treatment for patients with unstable type 1 diabetes mellitus. Although insulin independence can be achieved in these patients, persistence of normoglycemia is limited, while grafts are partially or fully rejected after a certain time even with multiple repetitions of the transplantation procedure [1]. Moreover, transplantation into the liver is often accompanied by several complications that may contribute to graft impairment. Therefore, various extrahepatic transplantation sites have been tested [2]; of these sites, artificial macroporous scaffolds have shown promising transplantation efficiency [3–6].

Non-invasive *in vivo* monitoring of transplanted islets can reveal the processes underlying islet engraftment and rejection through the assessment of islet viability, distribution, and mass. Precise monitoring by a reliable method may ultimately contribute to the improvement of transplantation outcomes. Visualization of transplanted islets requires labeling by a suitable contrast agent or genetic modification of isolated islets in order to obtain a specific signal from the transplanted islets in the host tissue. Various imaging modalities have been implemented for tracking transplanted islets, such as radionuclide methods [7, 8], magnetic resonance imaging (MRI) [9], optical imaging [10, 11], and ultrasound [12]. Each method provides different information and is affected by various limitations, such as low spatial resolution (radionuclide imaging), low specificity (proton (H-1) MRI), low sensitivity (fluorine (F-19) MRI), and signal attenuation (optical methods). Therefore, combining multiple imaging methods is desirable as it can provide more precise, complementary, and complete information.

The most widely used and clinically implemented MRI agents for islet labeling are superparamagnetic iron oxide nanoparticles (SPIONs) [13, 14] that are suitable for visualization by H-1 MRI. However, highly sensitive SPIONs possess low imaging specificity due to the presence of other *in vivo* sources of hypointense signals and thus are difficult to quantify. Fluorine-containing probes provide high specificity for *in vivo* detection by F-19 MRI due to the negligible amount of MR-detectable fluorine in biological tissues. Compared to other methods, the advantages of F-19 MRI are its high specificity, a similar resonance frequency to H-1, and the option for absolute quantification [15]. MRI requires a concentration in the order of millimolar for reliable detection. To achieve it in a cell implant, either high cellular uptake of the probe or probes containing more F-19 nuclei per molecule (*e.g.*, perfluorocarbons (PFC)) are required. Perfluoro-15-crown-5-ether (PFCE) with 20 equivalent fluorine nuclei is currently one of the mostly used PFC imaging agents [16]. PFCs are both hydrophobic and lipophobic synthetic compounds. Due to these properties, liquid PFCs are usually delivered as surfactant-stabilized PFC emulsions or, less often, entrapped in poly(lactic-*co*-glycolic acid) (PLGA) nanoparticles for F-19 MRI [17]. The encapsulation of PFC in PLGA nanoparticles leads to the further incorporation of various compounds, such as dyes, on the surface or inside of the nanoparticles, thus enabling multimodal imaging [18].

Recently, several studies reported on the application of F-19 probes for *in vivo* tracking of labeled cells [19–23], and also pancreatic islets [24, 25]. In the study by Barnett et al., human PIs were labeled with multimodal perfluorooctylbromide (for F-19 MRI, computed tomography and ultrasound) and transplanted under the kidney capsules of mice and rabbits. Although the transplanted islets were visualized *in vivo*, 2000 and 10,000 islets were needed to reach a sufficient signal-to-noise ratio (SNR) level at 9.4 T and 3 T respectively. Recently, 200 murine islets were transplanted and visualized at mouse thigh [25]. In this study and also in other cases of intracellular labeling, signal persistence in the tissue due to slow clearance of the label after cell death is a major issue [26, 27].

Although optical imaging involving fluorescence and bioluminescence (BLI) is a very sensitive method in contrast to F-19 MRI, it suffers from light scattering and absorption in biological tissues. Therefore, near-infrared (NIR) fluorescent dyes with lower attenuation are favorable for *in vivo* applications. The main advantage of BLI is the option to monitor islet viability *in vivo* [28]. However, it can be only implemented in experimental studies due to necessary genetic alteration of islets for luciferase expression.

In our previous study, we focused on optimizing a protocol for PI labeling using PLGA-based nanoparticles (PLGA-NPs) containing both PFCE and the NIR probe indocyanine green (ICG). PIs were visualized *in vitro* using F-19 MRI and fluorescence imaging [29]. The aim of the current study was to monitor the fate of PIs labeled with bimodal PLGA-NPs after transplantation into artificial scaffolds using trimodal imaging (F-19 MRI, fluorescence, and bioluminescence imaging). Importantly, the bioluminescence reporter constructor is a direct marker for PI viability and was used to validate the F-19 data *in vivo*. Together, the BLI and F-19 MRI data could address the main issue about clearance of probes from dead islets/cells and their contribution to false positives in the MRI data.

## Materials and Methods

All animal protocols were approved by the Ethics Committee of the Institute for Clinical and Experimental Medicine and the Ministry of Health of the Czech Republic (No. 58/2014) in accordance with the European Communities Council Directive (2010/63/EU).

### Isolation of Pancreatic Islets

Pancreatic islets were isolated from transgenic Lewis rats either with ubiquital expression of a gene for the luciferase enzyme (National BioResource Project – Rat, Kyoto, Japan) or from their non-bioluminescent littermates. Ten luciferase-negative (LUC−) animals were used as donors for *in vitro* examination of islets, while 20 luciferase-positive (LUC+) animals were used as donors of pancreatic islets for transplantation.

Isolation of pancreatic islets was performed according to a standard protocol described by Gotoh [30]. A culture medium containing 84 % CMRL-1066 medium, 10 % FBS, 5 % HEPES, 0.5 % penicillin/streptomycin (all Sigma-Aldrich, USA), and 0.5 % glutaMAX (Thermo Fisher Scientific, USA) was used throughout the study. After isolation, pancreatic islets were incubated (37 °C, CO_2_ atmosphere) in the culture medium overnight for recovery.

### Labeling of Pancreatic Islets

The bimodal PLGA-based nanoparticles were prepared using a single-emulsion solvent evaporation method as described previously [18]. Briefly, 100 mg of PLGA was dissolved in 3 ml dichloromethane. Nine hundred microliters of PFCE and 1 mg of indocyanine green was added to the organic phase. Next, the organic phase was added to the aqueous phase containing a surfactant under ultrasonication. To formulate positively charged particles, the protocol was slightly modified by adding 0.4 g of diethylaminoethyl-dextran (Sigma-Aldrich, Germany) to the aqueous phase. The size of the nanoparticles—measured using dynamic light scattering (DLS; Zetasizer Nano – Malvern Instruments Ltd., UK)—was 180 nm with a polydispersity index of 0.1. The PFCE content—measured on a Bruker Avance III 400 MHz NMR spectrometer (Bruker, Germany)—was 1.8 × 10^18^ fluorine atoms per milligram of the lyophilized sample. The bimodal nanoparticles (in the form of a freeze-dried powder) were resuspended in phosphate-buffered saline (PBS) before brief bath sonication.

For the *in vitro* experiment, the isolated PIs were labeled by endocytosis. The islets were incubated for 24 h (37 °C, 5 % CO_2_) in a culture medium containing 12 mg/ml, 17 mg/ml, and 23 mg/ml of the PLGA-NPs (500 islets for each concentration). They were then collected and washed three times with Hanks solution (Sigma-Aldrich, USA) after labeling. Control islets were incubated in a medium without nanoparticles and treated in the same way as the labeled islets. These islets were counted in a black well, handpicked, and subsequently fixed with 4 % formaldehyde.

For transplantation, the isolated islets were incubated for 24 h in the culture medium containing 17 mg/ml of PLGA-NPs, a quantity chosen based on the *in vitro* results. Control islets were incubated in a medium without nanoparticles. These islets were then washed three times with Hanks solution supplemented with 1 % fetal bovine serum (FBS), counted under a microscope, and placed in a plastic tube prior to transplantation.

### Viability and Functionality Assessment of Labeled Islets

Viability of labeled islets was assessed by staining with fluorescent dyes to reflect cell membrane integrity. Ten islets (in 20 μl of Hanks solution) were handpicked and placed in a well containing 9.4 μM of propidium iodide and 75 μM of acridine orange. After 5 min, 200 μl of PBS was added and the suspension was examined under a fluorescent microscope. Viability was expressed as the number of live (green) and dead (orange) cells. The ratio of live cells to all cells in the ten chosen islets was expressed as a percentage. Viability of labeled and unlabeled islets was also assessed by *in vitro* bioluminescence imaging.

The functional status of the labeled islets was tested using a glucose-stimulated insulin secretion test. Duplicates of 50 labeled and unlabeled islets were subsequently incubated (37 °C, 5 % CO_2_ atmosphere) in a basal Krebs medium containing low (3.3 mM), then high (22 mM) and low (3.3 mM) glucose concentrations again. After each incubation, an aliquot of the medium was taken and frozen at − 20 °C. Insulin content was then measured using an ELISA test. The amount of insulin released from the islets upon glucose stimulation was assessed as the glucose stimulation index representing the ratio of insulin content according to high and low glucose samples.

### Imaging Sensitivity Assessment of Labeled Islets

All optical images (bioluminescence and fluorescence) were acquired on an IVIS Lumina XR imager (Perkin Elmer, USA) and processed using Living Image software (Perkin Elmer, USA). MRI imaging was performed on a 4.7 T scanner (Bruker BioSpin, Germany) using a home-made dual H-1/F-19 surface single-loop circular radiofrequency (RF) coil with a diameter 4 cm.

To assess the minimum number of labeled islets detectable by bioluminescence, different numbers (10, 30, 50, 100, 300) of bioluminescent islets labeled with multimodal nanoparticles (endocytosis 17 mg/ml of PLGA-NP) as well as unlabeled islets were placed in wells containing the medium. The bioluminescent images were measured within a 1-min exposure time after the addition of D-Luciferin (0.15 mg/ml; Medesa, Czech Republic).

Estimation of F-19 MRI and fluorescence imaging sensitivity was performed using fixed labeled islets (endocytosis 12 mg/ml, 17 mg/ml, and 23 mg/ml of PLGA-NPs) placed in test tubes in different quantities (50, 100, and 300 islets).

*In vitro* MRI measurement: *T*_*2*_-weighted H-1 MRI images were acquired for reference using a turbo spin echo sequence with the following parameters: repetition time *TR* = 3000 ms, echo spacing *TE* = 12 ms, effective echo time *TE*_*eff*_ = 36 ms, turbo factor 8, spatial resolution of 0.19 **×** 0.19 **×** 2 mm^3^, number of acquisitions *NA* = 4, and scan time 1 min. Coronal F-19 MRI images were acquired using a turbo spin echo sequence with the following parameters: *TR* = 1000 ms, *TE* = 3.2 ms, *TE*_*eff*_ = 42.2 ms, turbo factor 32, spatial resolution 1.56 **×** 1.56 **×** 15 mm^3^, *NA* = 4096, and scan time 1 h 8 min. F-19 MRI images were interpolated from matrix 32 **×** 32 to 256 **×** 256, converted to false colors, and then co-registered with H-1 MRI images using ImageJ software (version 1.46r, National Institutes of Health, USA) [31]. SNR values were calculated from the manually outlined regions of interest (ROIs) placed on each sample, a reference and a noise region in the F-19 MRI images using ImageJ.

The fluorine content of the labeled islets (*F*_PI_) was calculated from the F-19 MRI images by comparing the signal of labeled islets *S*_PI_ to the signal of reference *S*_REF1_ (containing a known number of F-19 atoms *F*_REF1_ in the voxel). The agent uptake was calculated according to the formula:

1 $$ {F}_{\mathrm{PI}}=\frac{S_{\mathrm{PI}}}{S_{\mathrm{REF}1}\ast N}\ast {F}_{\mathrm{REF}1}, $$

where *N* represents the number of PIs in the sample and expressed as the number of F-19 atoms incorporated in one islet/endocrine cell (assuming that 1 islet contains approximately 1000 cells).

Fluorescence images of the same samples were acquired during a 2-s exposure using aperture (*f/stop*) 4 and binning 4. Fluorescence excitation was set at 745 nm and emission at 810–875 nm. ROIs of the same size were drawn around each tube and the emitted optical signal was expressed as the radiance efficiency ([photons/s/cm^2^/sr]/(μW/cm^2^)). Fluorescence images were overlaid on photographs for reference.

### Animal Model of Extrahepatic Pancreatic Islet Transplantation

Male Lewis rats (Velaz, Czech Republic) weighing 350–450 g (*n* = 3) were chosen as the recipients of the pancreatic islets. A surgical non-fluorescent mesh was shaped into rounded scaffolds, which served as an artificial transplantation site. During surgery, the animals were kept under inhalation anesthesia using isoflurane (5 % for induction, 2 % during the surgery). Two incisions were made in the abdominal area of the rats before subcutaneously implanting scaffolds into the cavities created using scissors (two scaffolds per one animal). The implanted scaffolds were supplemented with polytetrafluoroethylene rods completely filling the cavity to avoid obliteration of the internal scaffold space. The rods were removed and the cavities closed using small polytetrafluoroethylene plugs 1 week after scaffold implantation. Three days after, the pancreatic islets were transplanted into the exposed cavities using a Hamilton syringe supplemented with a thin plastic tube in order to ensure the slow controlled injection of a large volume of islets (avoiding syringe obstruction). The scaffolds were closed using the plugs after transplantation and the incisions were tightly closed using 5–0 Vicryl sutures (Ethicon, Johnson & Johnson Medical, Ltd., UK).

The animals received the labeled LUC+ islets (3000 and 1000 in each scaffold) or the unlabeled LUC+ islets (3000 and 1000) as controls.

### *In Vivo* Trimodal Imaging of Pancreatic Islets Transplanted into Artificial Scaffolds

Animals with transplanted islets were examined by bioluminescence, fluorescence, and F-19 MRI imaging on days 1, 4, 8, and 14 after islet transplantation using the same MRI scanner, RF coil, and optical imager for the *in vitro* study. The rats were shaved in the area of the scaffolds prior to imaging in order to eliminate scattering and attenuation of the optical signal. The rats were anesthetized by intramuscular anesthesia (ketamine 36 mg/kg and dexmedetomidine 0.08 mg/kg).

*In vivo* fluorescence imaging was performed based on the same parameters used for the phantom study with an exposure time of 60 s. Bioluminescence images were then acquired before and after intravenous administration of D-luciferin solution (70 mg/kg) with a 60-s exposure time and an open emission filter. Bioluminescent images were overlaid on photographs for co-registration of the bioluminescent signal. ROIs of the same size were carefully outlined around each scaffold before calculating the total radiance efficiency ([photons/s/cm^2^/sr]/(μW/cm^2^) for fluorescence and the total radiance (photons/s/cm^2^/sr) for bioluminescence.

MRI was performed after optical imaging. Axial and coronal *T*_*2*_-weighted H-1 MRI images were acquired using the same parameters used for the phantom study (except for a spatial resolution of 0.23 × 0.23 × 2 mm^3^ and a scan time of 2.5 min). The frequency was then adjusted to the F-19 signal of a reference (containing a suspension of the nanoparticles in water at concentration 30 × 10^18^ F-19 atoms/ml) placed in close proximity to the scaffolds. Axial F-19 MRI images were acquired using a turbo spin echo sequence with the following parameters: *TR* = 1500 ms, *TE* = 3.2 ms, *TEeff* = 42.2 ms, turbo factor 32, slice thickness 20 mm, *FOV* = 60 × 33 mm, spatial resolution 1.9 × 1.0 × 20 mm^3^, *NA* = 768 (scan time 19 min), or *NA* = 2048 (scan time 51 min). The slice was oriented perpendicular to the main axis of the scaffold, with the selected slice thickness, thus covering the signal from the whole scaffold. F-19 MRI images were interpolated from the acquired matrix 32 × 32 to 256 × 256, converted to false colors, and then co-registered with anatomical H-1 MRI images using ImageJ.

The number of engrafted islets *N*_TxPI_ was quantified from the F-19 MRI images by comparing the signal of the transplanted islets *S*_TxPI_ to the signal of reference *S*_REF2_ and by taking into account the amount of F-19 atoms in reference *F*_REF2_ and the agent uptake per one islet *F*_PI_ estimated in the *in vitro* study. The number of islets was then calculated as

2 $$ {N}_{\mathrm{TxPI}}=\frac{\frac{S_{\mathrm{TxPI}}}{S_{\mathrm{REF}2}}\ast {F}_{\mathrm{REF}2}}{F_{\mathrm{PI}}}. $$

### Histological Analysis

Two weeks after islet transplantation, the scaffolds were removed, fixed in 10 % neutral-buffered formalin, and embedded in paraffin blocks. Four-micrometer-thick paraffin sections were cut and routinely stained with hematoxylin and eosin (HE) and Verhoeff-Van Gieson elastin stain. Immunohistochemical detection of insulin (mouse monoclonal, MU029-UC, Biogenex, USA) and luciferase (mouse monoclonal, Luci 21 1-107, Novus Biologicals, USA) was performed on 4-μm-thick paraffin sections. The primary anti-insulin antibody was detected using Simple Stain MAX PO (MULTI) Universal Immuno-peroxidase Polymer anti-mouse, anti-rabbit Histofine (Nichirei Biosciences, Japan). Histofine Simple Stain Rat MAX PO (Nichirei, Japan) was used for detecting luciferase. Finally, visualization was performed using the Dako Liquid DAB+ Substrate-Chromogen System (Agilent, USA) and counterstaining with Harris’s hematoxylin. The slides were viewed using standard light microscopy (Olympus BX41).

### Statistical Analysis

Statistical analysis was conducted using GraphPad Prism 6.02 (GraphPad Software Inc., USA). Values in the graphs are reported as mean ± standard deviation. Coefficients of regression (*R*^2^) are presented based on the results of linear regression analysis.

## Results

### *In Vitro* Viability and Functionality of Labeled Islets

**Fig. 1:**
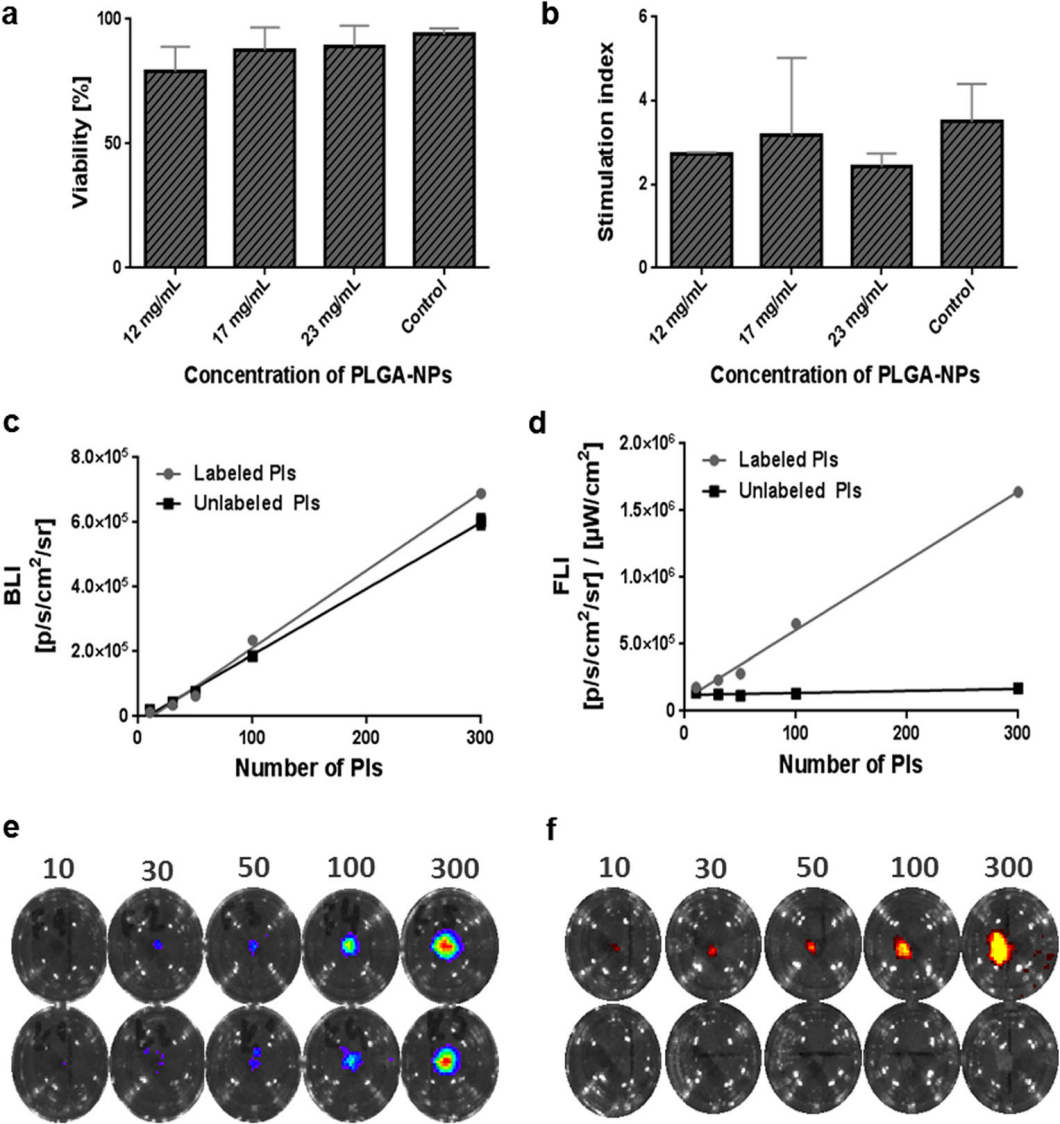
*In vitro* characterization of pancreatic islets. **a** Viability and **b** glucose stimulation indices of islets after labeling by endocytosis. Quantification of **c***in vitro* bioluminescence and **d** fluorescence signals from labeled and unlabeled islets. Representative **e** bioluminescence and **f** fluorescence images of different numbers of labeled (upper row) and unlabeled (bottom row) islets.

Nanoparticle labeling did not affect islet viability, while labeled islets showed comparable viability to unlabeled islets (Fig. 1a). Viability of the labeled islets was also confirmed by *in vitro* bioluminescence imaging, while labeled islets provided a similar bioluminescence signal (300 PIs—6.2 × 10^5^ p/s/cm^2^/sr) to unlabeled controls (300 PIs—6.0 × 10^5^ p/s/cm^2^/sr) (Fig. 1c, e).

Islet functionality after labeling was confirmed by measuring the insulin release upon glucose stimulation. Labeled islets had glucose stimulation indices above 2 (Fig. 1b).

### *In Vitro* Labeling and Visualization of Pancreatic Islets

Fluorescence imaging confirmed that unlabeled islets emitted no fluorescence signal after excitation at 745 nm (Fig. 1d, f). There was a linear relationship between the number of islets and their *in vitro* fluorescence and bioluminescence signals (both *R*^2^ = 0.99) (Fig. 1c, d).

**Fig. 2:**
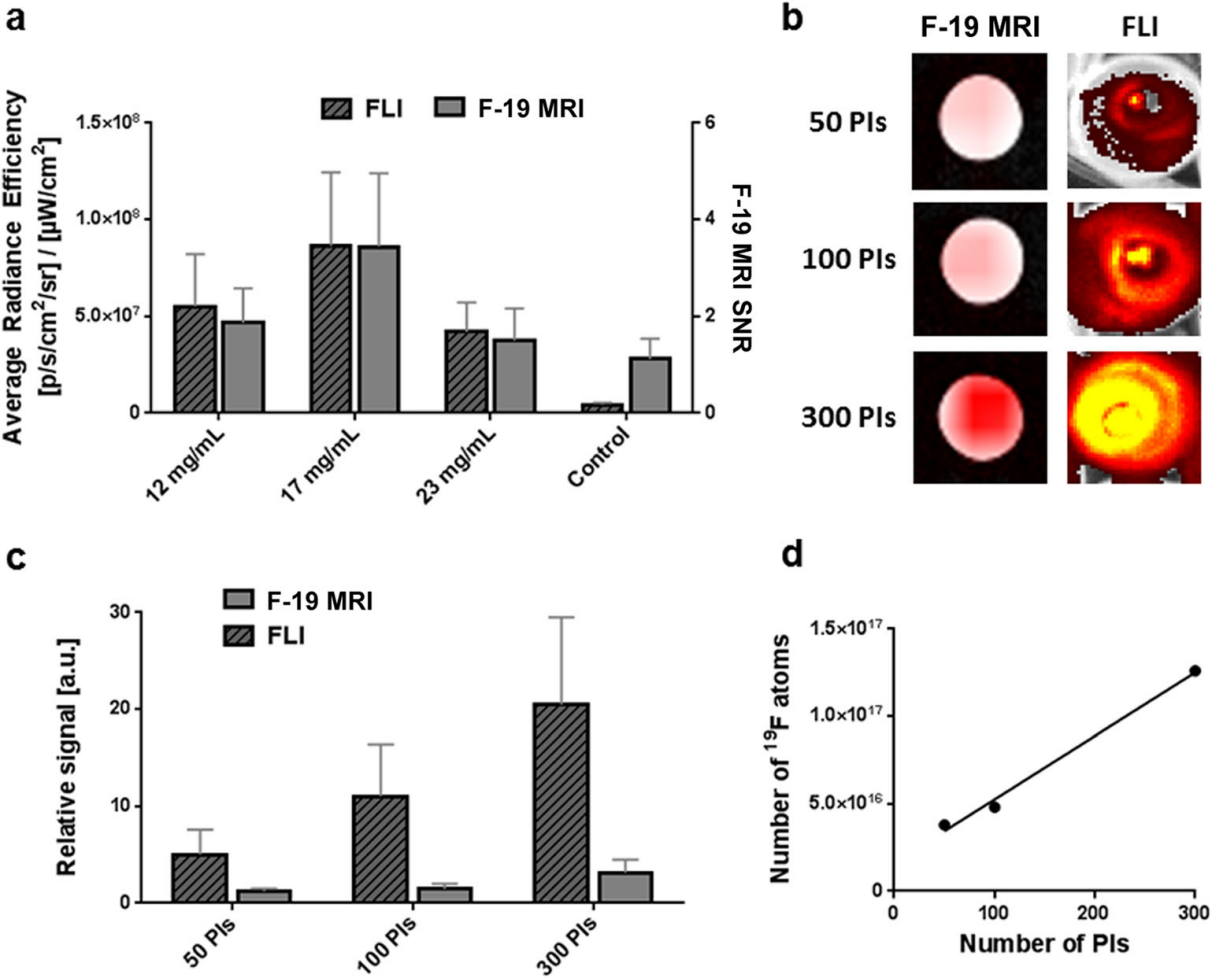
*In vitro* visualization of pancreatic islets labeled using endocytosis by F-19 MR and fluorescence imaging. **a** Comparison of signals originating from 300 islets labeled with different concentrations of nanoparticles. **b** Representative F-19 MR and FLI images of different numbers of islets labeled using 17 mg/ml of PLGA-NP. **c** Visualization sensitivity of various numbers of islets labeled at a 17 mg/ml concentration, where the relative signal is normalized to the signal from unlabeled islets. **d** Absolute quantification of the number of F-19 atoms incorporated in labeled islets.

As little as 50 islets were detected by F-19 MRI at an imaging time of 68 min. The highest F-19 MRI and fluorescence signals originated from islets labeled by simple co-incubation with 17 mg/ml of PLGA-NPs for 24 h (Fig. 2a, b). Fluorescence imaging of labeled islets provided a higher signal-to-noise ratio within substantially shorter scanning time than F-19 MRI. We therefore conclude, as expected, that fluorescence imaging is a more sensitive detection method (Fig. 2c).

*In vitro* F-19 MRI revealed incorporation of an average of 5.5 ± 1.8 × 10^14^ of F-19 per islet (approximately 5.5 ± 1.8 × 10^11^ of F-19 atoms per cell) when labeled with 17 mg/ml of PLGA-NPs (Fig. 2d).

### *In Vivo* Imaging of Transplanted Islets Using an Animal Model

**Fig. 3:**
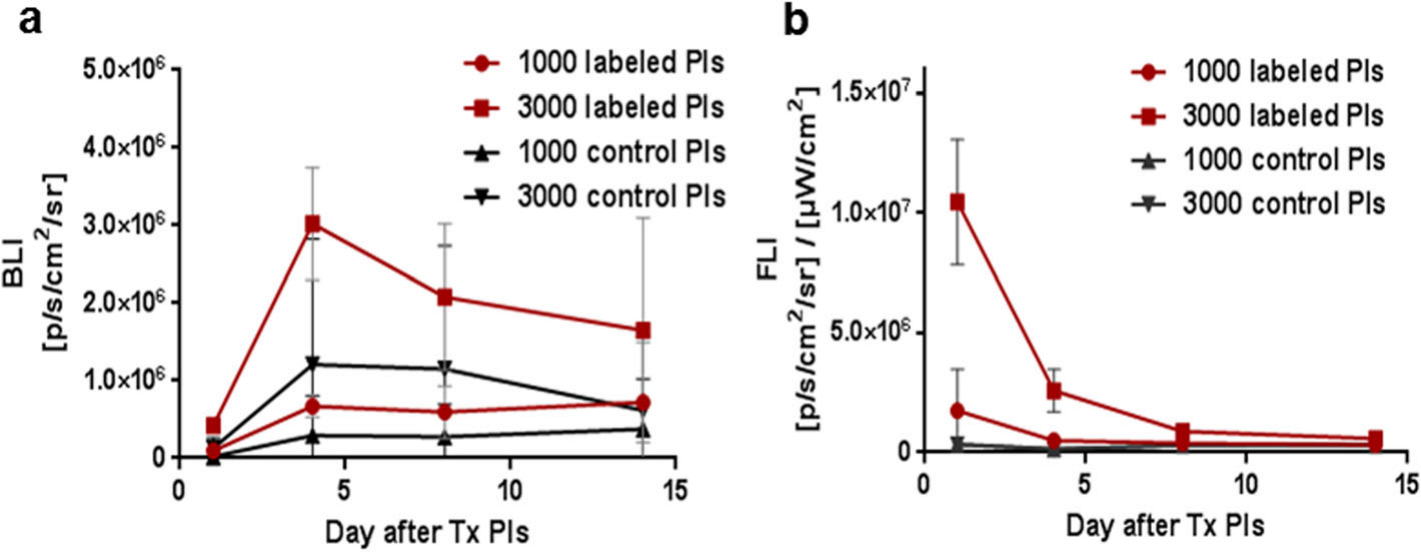
Quantification of optical signals from transplanted islets. The time course of **a** bioluminescence (BLI) and **b** fluorescence (FLI) signals originating from labeled and unlabeled islets.

*In vivo* BLI confirmed the presence of viable transplanted islets in the scaffolds throughout the entire 14-day experiment (Fig. 3a). Nanoparticle labeling did not impair the viability or survival of transplanted islets measured by *in vivo* bioluminescence, as the labeled islets provided a similar bioluminescence signal compared to unlabeled controls. Both labeled and unlabeled islets showed maximum bioluminescence on day 4 after transplantation, with the signal decreasing slightly by day 14 (55 % of that on day 4).

The fluorescence signal originating from the labeled islets reached its maximum immediately after transplantation (day 1), before rapidly decreasing over the next week in all experimental groups (Fig. 3b). The fluorescence signal measured on day 1 and day 4 decreased by 73 %, while unlabeled islets emitted no fluorescence signal at any point.

**Fig. 4:**
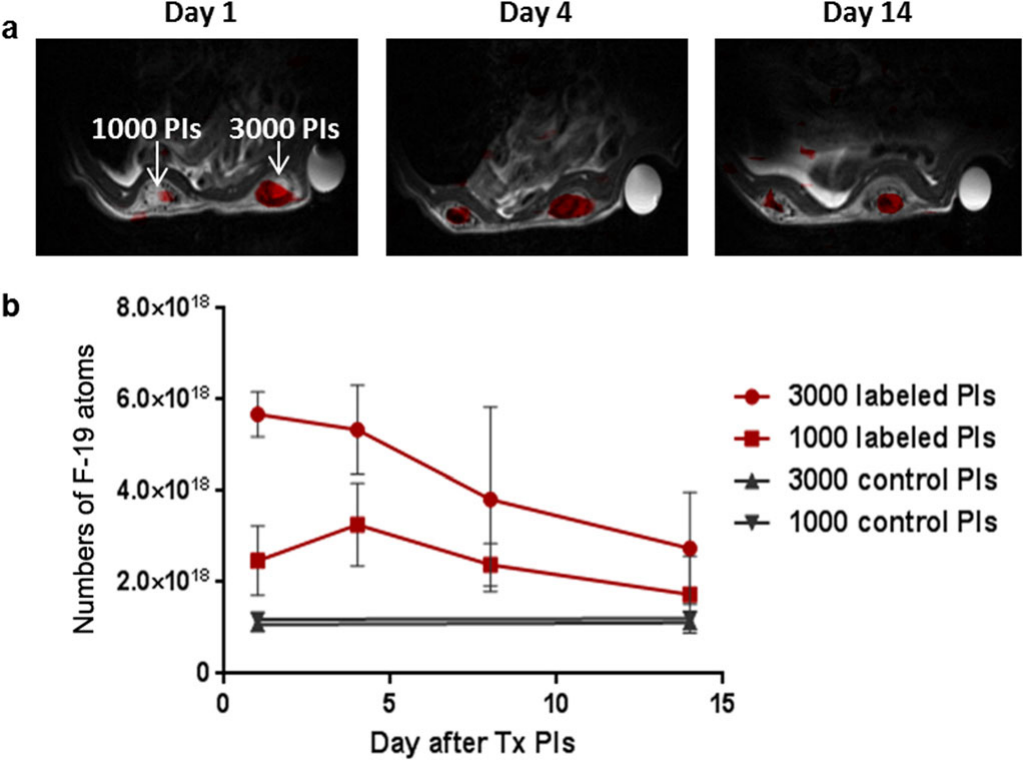
F-19 MRI of transplanted islets. **a** Representative F-19 MR images of pancreatic islets labeled with PLGA-NPs in artificial scaffolds. **b** Quantification of the F-19 MR signal from labeled islets.

The localization of labeled islets inside the scaffolds was also confirmed by F-19 MRI imaging throughout the whole long-term examination (Fig. 4a). The absolute signal revealed engraftment of an average of 2300 ± 200 and 1100 ± 300 islets in the scaffolds on day 1, corresponding to 3000 and 1000 transplanted islets respectively (manually counted prior to transplantation). The maximum F-19 MRI signal was detected on the first day after islet transplantation; after which, the signal continuously declined. However, the F-19 MRI signal originating from 1000 islets on day 14 was still above the noise level (Fig. 4b). The signal based on 3000 islets decreased to 66 % of the starting value on day 8 and to 47 % on day 14.

**Fig. 5:**
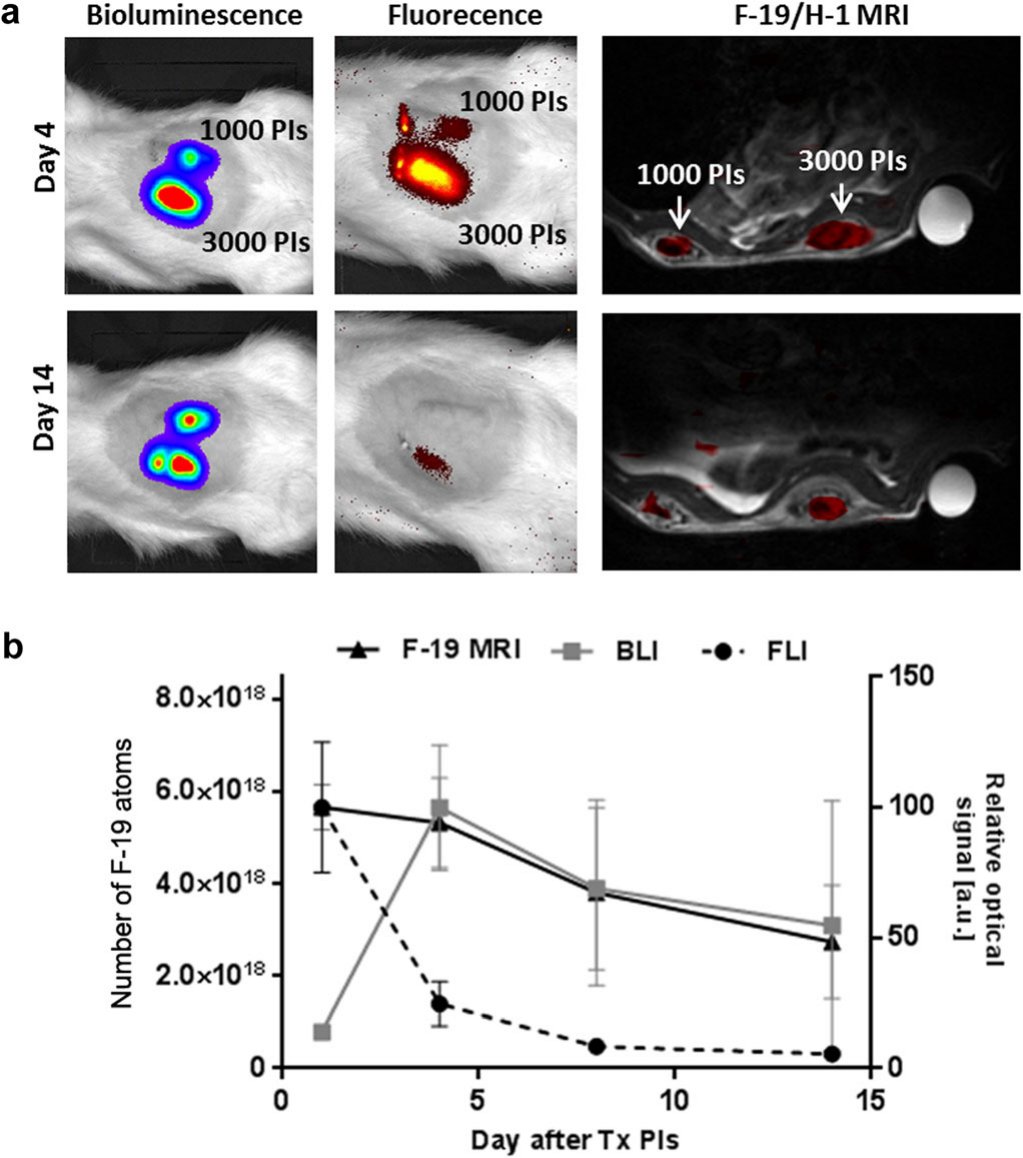
Trimodal imaging of transplanted pancreatic islets in scaffolds. **a** Representative bioluminescence, fluorescence, and axial F-19/H-1 MR images of 3000 and 1000 pancreatic islets transplanted into scaffolds on days 4 and 14. **b** Time course of bioluminescence (BLI), fluorescence (FLI), and F-19 MRI signals for 3000 labeled transplanted islets. MRI signal is recalculated to the corresponding number of F-19 nuclei (left axis); the optical signals (BLI, FLI) are normalized to the maximum value (= 100 %, right axis).

The slow decline of the BLI and F-19 MRI signals contrasted with the rapid decrease of FLI (Fig. 5). The F-19 MRI signal strongly correlated with bioluminescence between days 4 and 14 (*R*^2^ = 0.99).

### Histology

**Fig. 6:**
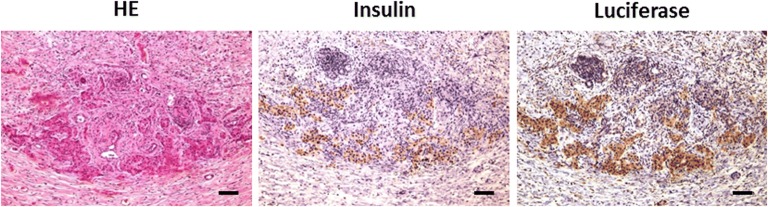
Histology of scaffolds on day 14 after islet transplantation. Images of a transplanted graft with pancreatic islets stained by hematoxylin/eosin (HE) and immunohistochemically with the primary antibodies anti-insulin and anti-luciferase. Insulin- and luciferase-positive cells were present at the same locations within the graft. Scale bars correspond to 100 μm.

Two weeks after islet transplantation, the rats were sacrificed and scaffolds subjected to histological examination. Viable vascularized pancreatic islets distorted by fibrosis were present in the central parts of the scaffolds. Irregularly distributed clusters of cells co-expressing insulin and luciferase were detected immunohistochemically in all islets (Fig. 6). Cells expressing both markers were arranged in trabeculae and occasional small ductular structures. Deposits of hemosiderin and the foreign body granulomatous reaction composed of macrophages and multinucleated foreign body giant cells were detected in some islets.

## Discussion

In this study, we used multimodal imaging to track pancreatic islets transplanted into subcutaneously implanted artificial scaffolds. This site possesses some benefits over the liver [3] as less invasive surgery, the possibility of removal of the whole graft in the case of complications (*e.g.*, rejection, inflammation), direct application of drugs, and the possibility of local enhancement of vascularization (*e.g.*, using the vascular factors/stem cells). Moreover, the scaffolds are suitable for examining transplanted cells using various imaging methods [4, 32]. In addition to the grafted islets being concentrated in one place, which is advantageous for *in vivo* imaging detection, the scaffolds are implanted subcutaneously, ensuring a short optical path for the fluorescence/bioluminescence signal emitted from the transplanted islets.

Labeling by endocytosis maintained the viability and functionality of the labeled islets, which was also proved by a comparable bioluminescence signal between labeled and unlabeled islets. Sufficient labeling of islets *via* endocytosis using PLGA-NPs was also confirmed by confocal microscopy in our previous paper [29]. Since the bioluminescence signal is dependent on the presence of oxygen and adenosine triphosphate, only viable cells emit photons in a bioluminescence reaction [28]. Published studies on the following topics attest to the high viability and insulin secretion of labeled islets: non-toxicity of probes based on perfluorocarbons [33] and the safety of selected labeling methods [29]. The same nanoparticles have been used to label various subsets of primary human dendritic cells in preparation for clinical application [34]. Thus, the clinical application of this procedure is feasible.

*In vitro* F-19 MRI and fluorescence imaging confirmed the efficiency of our labeling procedures. Using endocytosis, the highest FLI and MRI signals were detected from islets labeled with 17 mg/ml of PLGA-NPs, whereas incubation at a concentration of 23 mg/ml produced a worse outcome. We speculate that the positively charged fluorine nanoparticles could aggregate on the islet surface after long incubation times if the high concentration of PLGA-NPs (≈ 23 mg/ml) was used. This excess of nanoparticles could limit further agent uptake. Nevertheless, labeling using 17 mg/ml of PLGA-NPs led to the incorporation of 5.5 ± 1.8 × 10^14^ of F-19 per islet (approximately 5.5 ± 1.8 × 10^11^ of F-19 nuclei), a finding that accords with published data (range of 10^11^–10^13^) [35–37]. The nanoparticles have been previously shown to locate intracellularly in different cell types [16, 17].

*In vitro* bioluminescence and fluorescence signals originating from labeled islets correlated with the number of islets, which points to their reliability for quantification of transplanted mass (under *in vitro* conditions or immediately after transplantation). Although, 50 islets were detected by *in vitro* imaging, a much higher amount is needed for adequate visualization under *in vivo* conditions due to the dispersion of islets in the scaffolds and scattering and absorption of the optical signal by the tissue.

Transplanted islets were visualized in artificial scaffolds by bioluminescence, fluorescence, and F-19 MRI imaging over 2 weeks. Bioluminescence confirmed the viability of islets in the scaffolds throughout the whole examination period with only a partial decrease in islet mass. Absolute quantification from F-19 MRI images confirmed appropriate numbers of transplanted islets in scaffolds on day 1 after islet transplantation. Both 1000 and 3000 islets were detectable by F-19 MRI for the whole examination (14 days). The decrease of the F-19 MRI signal to 44 % on day 14 corresponds with published experimental and clinical data reporting a gradual loss of transplanted islets over 2 weeks after transplantation [14, 38]. Previous studies have visualized islets labeled by fluorine-containing probes at one time point post-transplantation only [24, 39] or for several weeks [25]. The recent study reported a long-term monitoring of transplanted islets using F-19 MRI, fluorescence, and bioluminescence imaging within 70 days. While the F-19 MRI and FLI signals were decreasing slowly, the BLI signal gradually decreased until day 7 (approximately by 80 %), which means that either dead cells with labels or free released labels stayed at the site of transplantation. Similar effect was observed also in other studies with fluorine-labeled cells resulting to false-positives [16, 21, 27]. In our model, viability of islets decreased only by 33 % within 14 days after transplantation, which suggest that our transplantation and imaging model better reflects the status of transplanted islets. Therefore, our transplantation model together with the possibility of its monitoring by F-19 MRI has a greater potential for application in clinical practice. Moreover, sufficient vascularization is crucial for islet survival and it should be superior in the case of polymer scaffolds compared to the muscle.

It should be noted that due to low sensitivity of F-19 MRI, long acquisition times (1 h) were needed to visualize the transplanted grafts in our study. Low spatial resolution has been used to improve sensitivity, but it can also lead to the sizes of small grafts being underestimated due to the spreading of the signal in the image voxel [33]. Low detection sensitivity of *in vivo* F-19 MRI has been also reported in other models, *e.g.*, examination of carbohydrates sensitive to beta cells through GLUT-2 transporters in order to visualize transplanted islets [40] and tracking transplanted stem cells in the brain [33]. However, the ability to quantify signals and estimate graft size without the use of radioactive probes represents a considerable advantage.

Inhomogeneous *B*_*1*_ excitation when using the surface RF coil is a further limitation. The F-19 MRI signal is influenced not only by F-19 concentration, but also by the distance from the coil. Other errors in quantification of the F-19 MRI signal may arise from filtering during post-processing, low measurement matrices, and Fourier transform, potentially resulting in partial signal dispersion within the whole measurement matrix [29, 41]. To overcome these sensitivity problems and to improve F-19 SNR, various data acquisition methods, such as compressed sensing [39] and ultrashort echo sequences [42], have been proposed, which could reduce imaging time while maintaining or even improving the SNR. These advanced methods were not available for our imaging system. Nevertheless, we have shown here that the technique has sufficient sensitivity even without optimal imaging sequences.

Although fluorescence imaging was found to be a more sensitive method for cell tracking compared to F-19 MRI, we observed a steep decline in the *in vivo* fluorescence signal. Quenching of the fluorescence signal originating from islets labeled by the high concentration of PLGA-NPs has been described previously [29], but the fluorescence signal used in this model decreased rapidly within 4 days after transplantation. Fluorescence of labeled islets decreased to a noise level within 1 week, while F-19 MRI and bioluminescence signals decreased only partially. This indicates the instability of the fluorescent dye (ICG) in the nanoparticles under *in vivo* conditions. Thermal degradation of ICG in other multifunctional perfluorocarbon nanoemulsions at temperatures above 37 °C, which results in decreased light absorption and decreased fluorescence, has been previously reported [43]. Alternatively, the dye may leak out of the islets over time [17]. To confirm the instability of the ICG dye in the nanoparticles, we performed an *in vitro* experiment with long-term incubation of the nanoparticles at various pH (5.8 and 7.4) and temperatures (25 °C and 37 °C). We observed a decrease of the fluorescence signal originating from the particles over time (see Electronic Supplementary Material (ESM)). Moreover, the strong fluorescence signal from the supernatant was detected, which confirmed a substantial release of ICG from the particles within the first 4 days (Fig. S1b ESM). The release of ICG from the particles together with photobleaching over time limits the reliability of longitudinal *in vivo* fluorescence imaging with this probe.

The bioluminescence signal we observed correlated strongly with the F-19 MRI signal from the transplanted islets, which indicates that the probe was washed out after cell death. This finding is in apparent contradiction to a previously published study that used PFC-labeled neural stem cells [33]. The authors of that study found that the agent remained in the tissue, while the F-19 MRI signal persisted even after cell death in the case of stem cells transplanted into brain tissue. Another study, one that also used lipid-coated emulsions, found that perfluoro-crown-ether was retained at the site in a murine model of inflammation for several months [44]. It should also be noted that the F-19 labels used in these studies were different (lipid-coated emulsion *versus* PLGA nanoparticles). Moreover, in the study of Liang et al., the results of BLI and F-19 MRI substantially differed suggesting a similar effect [25]. Different formulation may also significantly influence the PFC clearance rate. Furthermore, the islets in our study were transplanted into a well-vascularized site with good access to circulating macrophages. We hypothesize that migrating macrophages removed the nanoparticles together with the remnants of the dead islets, thus eliminating their contribution to any false-positive results. In any case, clearance from dead cells is essential for avoiding false-positives, a major issue that we resolved through the use of PLGA-NPs.

Finally, histology revealed viable islet grafts in scaffolds containing both labeled and unlabeled islets. It is significant that insulin deposits were found at the same locations as luciferase molecules, which confirmed the functionality of transplanted LUC+ islets labeled with PLGA-NPs.

## Conclusion

We present a novel platform for *in vivo* multimodal tracking of transplanted pancreatic islets. Using three different imaging methods, we obtained complementary information on graft localization, size, and viability. The model presented here may provide insights into the processes connected with the engraftment and rejection of transplanted pancreatic islets in a non-invasive and kinetic manner and be of help to future *in vivo* studies. Although fluorescent imaging of the ICG dye in PLGA-based nanoparticles was also very sensitive shortly after transplantation, the instability of the dye under longitudinal *in vivo* examination represents substantial issues. Bioluminescence imaging confirmed the viability of the transplanted islets throughout the whole experiment. Importantly, the quantitative F-19 signal correlated strongly with islet viability, which indicates that the PLGA-NPs are cleared from dead islets, thus eliminating any false-positives from the F-19 MRI data.

## Electronic Supplementary Material

ESM 1 (DOCX 102 kb)
